# Scientific output quality of 40 globally top-ranked medical researchers in the field of osteoporosis

**DOI:** 10.1007/s11657-018-0446-4

**Published:** 2018-03-26

**Authors:** W. Pluskiewicz, B. Drozdzowska, P. Adamczyk, K. Noga

**Affiliations:** 1 Department and Clinic of Internal Diseases, Diabetology and Nephrology, Metabolic Bone Diseases Unit, School of Medicine with the Division of Dentistry in Zabrze, Medical University of Silesia, Katowice, Poland; 2 Department and Chair of Pathomorphology, School of Medicine with the Division of Dentistry in Zabrze, Medical University of Silesia, Katowice, Poland; 3 Department of Paediatrics, School of Medicine with the Division of Dentistry in Zabrze, Medical University of Silesia, Katowice, Poland; 4 Main Library, Medical University of Silesia, Katowice, Poland

**Keywords:** Citations, *H*-index, Individual output, Osteoporosis, Science quality index

## Abstract

**Summary:**

The study presents the research output of 40 globally top-ranked authors, publishing in the field of osteoporosis. Their *h*-index is compared with the Scientific Quality Index (SQI), a novel indicator. Using SQI, 92.5% of the authors changed their initial positions in the general ranking. SQI partially depends on bibliometric measures different from those influencing *h*-index and may be considered as an assessment tool, reflecting more objective, qualitative, rather than quantitative, features of individual scientific output.

**Purpose:**

The study approaches the research output of 40 globally top-ranked authors in the field of osteoporosis.

**Methods:**

The assessed authors were identified in the Scopus database, using the key word “osteoporosis” and the *h*-index data, collected during the last decade (2008–2017). The data, concerning the scientific output, expressed by the *h*-index, were compared with a novel indicator of scientific quality—called the Scientific Quality Index (SQI). SQI is calculated according to the following formula: Parameter No. 1 + Parameter No. 2, where: Parameter No. 1 (the percent of papers cited ≥ 10 times) the number of papers cited ≥ 10 times (excluding self-citations and citations of all co-authors) is divided by the number of all the published papers (including the papers with no citation) × 100%, Parameter No. 2 (the mean number of citations per paper) the total number of citations (excluding self-citations and citations of all co-authors) divided by the number of all published papers (including papers with no citation).

**Results:**

The following research output values were obtained: the citation index, 2483.6 ± 1348.7; the total number of papers, 75.1 ± 23.2; the total number of cited papers, 69.3 ± 22.0; the number of papers cited, at least, 10 times, 45.4 ± 17.2; the percent of papers cited, at least, 10 times, 59.9 ± 10.0; and the mean citations per paper, 32.8 ± 15.0. The mean value of Hirsch index was 24.2 ± 6.2 and SQI 92.7 ± 22.3. Using SQI, only three authors did not change their initial ranking position, established according to the *h*-index; 18 authors noted a decrease, while other 19 improved their initial ranking position. The *h*-index correlated with SQI; *r* = 0.72; *p* < 0.0001.

**Conclusion:**

Qualitative features of scientific output, reflected by SQI, have changed the classification of 92.5% of authors. SQI may be considered as an assessment tool which is more strongly determined by qualitative than quantitative features of individual scientific output.

## Introduction

The are several methods to assess scientific research output of an individual scientist. Traditionally, the scientific output can be measured by the number of papers, the number of papers in which a given author is either the first or a senior author, the total citation index, the citation index after exclusion of self-citations, or the citation index after exclusion of citations of all co-authors. These criteria should be considered as parameters, describing mainly the quantitative features in the evaluation of personal output, while its quality still remains largely uncharted. The idea of evaluating both the quantity and quality of scientific activity was developed by Hirsch [1]. He proposed an original, simple indicator to characterize the cumulative impact of the research work of individual scientists: a scientist has got *h*-index if *h* of his/her *N* papers have at least *h* citations each, and the other (*N*-*h*) papers have no more than *h* citations each. The Hirsch index (*h-*index) has been widely used during the last decade by scientific societies worldwide. In several studies, this index was discussed and compared with other methods of personal contribution assessment in research [2–10]. Although, so far, the *h-*index is used to be considered as the best indicator of individual contribution to science, it is noticeable that its personal value is influenced both by the effects of quantity (the number of publications) and quality (the number of citations).

Recently, a novel indicator of scientific individual output has been developed [11]. The Scientific Quality Index (SQI) has been defined, according to the following formula: [the percent of papers cited ≥ 10 times vs. all the published papers, including those with no citation] + [the mean number of citations per paper, regarding all the published papers, including those with no citation]. Self-citations and citations of all co-authors are excluded. The detailed equation to calculate SQI is given in the “Methods” section. The SQI formula clearly indicates SQI to be strongly influenced by highly cited papers, so it is expected that SQI, in comparison to the *h-*index, should be more related to science quality but less dependent on the overall quantity of publications.

The aim of the current study was to present the research output of 40 globally top-ranked world authors in the field of osteoporosis and to compare their classification, based on *h-*index and SQI criteria.

## Methods

Data for researchers, active in the field of osteoporosis with the highest *h-*index values for the last decade (2008–2017), were derived from Scopus (the Elsevier’s largest abstract and citation database of peer-reviewed literature). In case of the same value of the *h-*index, the position in the ranking list was established, on the basis of the citation index. Only the papers, related directly to the field of osteoporosis, were taken into consideration. In case of the authors publishing in different areas of medical research, only their contribution to the field of osteoporosis was taken into account. The *h-*index-based assessment of the scientific output of such selected authors was juxtaposed with SQI values.

According to authors’ opinion, the basic SQI characteristics may be provided by pointing out its following traits: *first*, it is easy to calculate; *second*, the analyzed data are available in a commonly used database; and *third*, the index expresses the scientific output’s quality of an individual researcher.

The following data were collected: *h-*index for all citations; the citation index, except the citations by the author and of all co-authors; the number of all published papers; the number of cited papers; and the number of papers, cited at least 10 times and the percent of these papers vs. all the published papers, including those with no citation. The threshold of 10 citations was employed, following an assumption that ≥ 10 citations indicate a fairly significant attention from other researchers of the international scientific community. The ratio of ≥ 10 times cited papers to all the published papers may thus be approached as a quality index of the entire scientific output of a given author.

SQI was calculated according to the following formula: Parameter No. 1 + Parameter No. 2, where:

Parameter No. 1 (the percent of papers cited ≥ 10 times): the number of papers cited ≥ 10 times (excluding self-citations and citations of all co-authors) divided by the number of all the published papers (including the papers with no citation) × 100%,
Parameter No. 2 (the mean number of citations per paper): the total number of citations (excluding self-citations and citations of all co-authors) divided by the number of all published papers (including papers with no citation).

The data were collected on 2nd November 2017.

## Statistics

All the calculations were done using the Statistica software (StatSoft, Tulsa, OK, USA). Descriptive statistical values were presented as mean values and standard deviations. The normality of distribution of analyzed data was checked by the Shapiro-Wilk test. A correlation analysis was performed using Pearson’s correlation coefficient, and coefficients of correlation were compared by the Fisher exact test.

## Results

Table 1 presents assessed scientific output data of individual authors. The mean research outputs were as follows: the citation index, 2483.6 ± 1348.7; the total number of papers, 75.1 ± 23.2; the total number of cited papers, 69.3 ± 22.0; the number of ≤ papers cited ≤ 10 times, 45.4 ± 17.2; the percent of papers cited ≥ 10 times, 59.9 ± 10.0%; and the mean number of citations per paper, 32.8 ± 15.0. The mean value of the Hirsch index was 24.2 ± 6.2 and SQI was 92.7 ± 22.3.

**Table 1:** Individual scientific output

Author’s name	Number of publications 2008–2017	The *h*-index without citations of all co-authors	Citations without citations of all co-authors	No. of all cited papers	No. of papers cited ≥ 10 times	Percent of papers cited ≥ 10 times (Parameter No. 1)	No. of mean citations per paper (Parameter No. 2)	SQI	*H*-index-based position	SQI-based position	Change
S. Boonen	93	36	3889	89	73	78.5	41.8	120.3	1	6	− 5
S. Cummings	87	35	5311	86	72	82.7	61	143.7	2	1	+ 1
R. Eastell	98	35	5579	91	63	64.3	56.9	121.1	3	5	− 2
J. Kanis	111	35	5227	105	73	65.8	49.8	115.6	4	7	− 3
C. Roux	118	35	3714	111	80	67.8	31.5	99.3	5	15	− 10
C. Cooper	119	32	4504	109	68	57.1	37.8	94.9	6	18	− 12
J. Adachi	120	32	2623	111	72	60	21.8	81.8	7	27	− 20
J. Cauley	122	30	3790	112	81	66.4	31.1	97.5	8	17	− 9
J. Reginster	105	30	3292	95	63	60	31.3	91.3	9	21	− 12
K. Ensrud	88	30	2612	85	65	74.9	29.7	104.6	10	11	− 1
E. McCloskey	89	27	4578	84	52	58.4	51.4	109.8	11	9	+ 2
P. Miller	65	27	2433	64	43	66.1	37.4	103.5	12	12	0
K. Saag	93	26	2487	83	52	55.9	26.7	82.6	13	26	− 13
S. Khosla	55	25	3012	54	40	72.7	54.8	127.5	14	4	+ 10
E. Lewiecki	66	25	2652	58	36	54.5	40.2	94.7	15	19	− 4
W. Leslie	119	25	2321	113	63	52.9	19.5	72.4	16	30	− 14
R. Rizzoli	68	25	2242	57	39	57.4	33	90.4	17	23	− 6
J. Brown	63	25	2078	61	46	73	33	106	18	10	+ 8
H. Johansson	68	24	3311	63	43	63.2	48.7	111.9	19	8	+ 11
I. Reid	55	23	4273	52	32	58.2	77.7	135.9	20	2	+ 18
J. Eisman	48	23	2875	46	34	70.8	59.9	130.7	21	3	+ 18
D. Hanley	50	23	1530	50	35	70	30.6	100.6	22	14	+ 8
R. Recker	65	23	1878	64	45	69.2	28.9	98.1	23	16	+ 7
S. Adami	62	22	2622	57	37	59.7	42.3	102	24	13	+ 9
S. Silverman	74	22	2138	65	46	62.2	28.9	91.1	25	22	+ 3
M. Brandi	77	22	1983	67	34	44.2	25.7	69.9	26	32	− 6
N. Binkley	56	21	1612	52	34	60.7	28.8	89.5	27	24	+ 3
S. Greenspan	57	21	1484	55	39	68.4	26	94.4	28	20	+ 8
A. Papaioannou	74	21	1205	67	41	55.4	16.3	71.7	29	31	− 2
N. Watts	54	20	1370	51	31	57.4	25.4	82.8	30	25	+ 5
J. Curtis	61	20	1141	53	30	49.2	18.7	67.9	31	35	− 4
P. Leung	61	18	1646	56	33	54.1	27	81.1	32	28	+ 4
M. Rossini	66	18	1114	59	30	45.5	16.9	62.4	33	38	− 5
P. Hadji	70	17	1347	57	28	40	19.2	59.2	34	39	− 5
T. Nakamura	58	17	939	54	30	51.7	16.2	67.2	35	37	− 2
M. Shiraki	62	17	899	56	33	53.2	14.5	67.7	36	36	0
T. Sugamoto	56	16	1035	44	28	50	18.5	68.5	37	33	+ 4
K. Brixen	47	16	1019	44	26	55.3	21.7	77	38	29	+ 9
L. Lix	54	15	897	49	28	51.8	16.6	68.4	39	34	+ 5
M. Amling	49	14	680	43	19	38.8	13.9	52.7	40	40	0

Column 9 of Table 1 shows the researcher’s position in the *h-*index ranking with descending order. In case of the same *h-*index values, the position was established by the citation index. Column 10 presents the researcher’s position in the SQI-based ranking and the last column shows SQI-based position changes versus corresponding positions expressed in the *h-*index-based ranking.

Using SQI, only three authors did not change their initial *h-*index positions, while 18 authors demonstrated a decrease and 19 improved their initial position. The greatest drop was identified for the author with the 7th position in the *h-*index-based ranking (− 20 to position 27). The highest improvement (by 18 positions) was observed for the authors classified as the 20th and the 21st, while in the SQI assessment, those two authors were ranked as the 2nd and the 3rd, respectively.

**Fig. 1:**
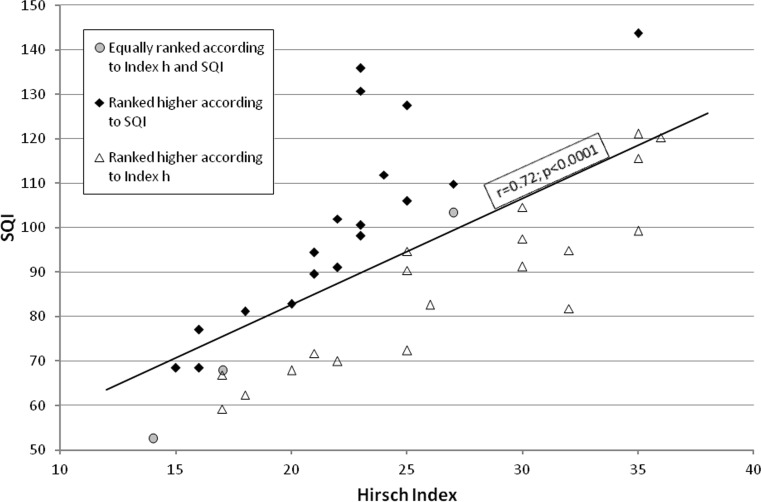
Correlation between the *h*-index and SQI

*H-*index and SQI values for particular researchers are presented in Fig. 1. The *h-*index correlated significantly with SQI; *r* = 0.72; *p* < 0.0001.

Some information on the role of basic bibliometric parameters, influencing the *h-*index and SQI, may be derived from correlation analyses (see Table 2). The *h-*index is significantly correlated with all the presented parameters. SQI does not depend on two purely quantitative factors, e.g., the number of all publications and the number of all cited papers. The lack of significant influence of the number of publications and of the number of cited papers on SQI indicates that quantity plays a weaker role for this indicator, whereas the impact of these two factors on the *h-*index is fairly strong. The number of papers cited ≥ 10 times, which is still a factor quantitative rather than qualitative in character, significantly correlated with both indices, however, more with the *h-*index, while the two, mainly qualitative parameters, e.g., the percent of papers cited ≥ 10 times (SQI Parameter No. 1) and the mean number of citations per paper (SQI Parameter No. 2), have a much stronger influence on SQI than on the *h-*index. The total number of citations equally influenced both the *h-*index and SQI.

**Table 2:** Correlation analysis of the *h*-index and SQI

Bibliometric parameter	Correlated with:		*p* value^#^
	*H*-index	SQI	
Number of publications	0.74 (*p* < 0.0001)	0.17 (NS)	< 0.001
Number of citations	0.90 (*p* < 0.0001)	0.83 (*p* < 0.0001)	NS
Number of cited papers	0.80 (*p* < 0.0001)	0.29 (NS)	< 0.001
Number of papers cited ≥10 times	0.93 (*p* < 0.0001)	0.59 (*p* < 0.0001)	< 0.0001
Percent of papers cited ≥10 times	0.67 (*p* < 0.0001)	0.85 (*p* < 0.0001)	< 0.05
Mean citations per paper	0.71 (*p* < 0.0001)	0.94 (*p* < 0.0001)	< 0.001

^#^*p* value for comparison between two coefficients of correlation

## Discussion

A novel approach to the assessment of scientific achievements, presented in the reported study, offers a new interpretation of the term “quality” with regard to individual scientific research output. The majority of globally top authors in the field of osteoporosis changed their *h-*index-based ranking position after SQI ranking was applied. One may then assume that for a significant part of researchers, their scientific output, evaluated by a mixed qualitative/quantitative variable, such as the *h-*index will differ from ranking figures, based on a more qualitative variable, such as SQI. This is the most important finding of the current study.

In general, the SQI-based classification is more closely related to Parameter No. 1, e.g., the percent of papers, cited ≥ 10 times. Approximately, $$ \raisebox{1ex}{$2$}\!\left/ \!\raisebox{-1ex}{$3$}\right. $$ of total SQI value was dependent on Parameter No. 1, and the mean number of citations per paper, being a more “quantitative” parameter, was less important. Therefore, it may be assumed that SQI is stronger and more objective than the *h-*index, regarding the actual quality of individual research output. Only one author demonstrated a higher value of Parameter No. 2 than that of Parameter No. 1.

In our previous study [11], which was the first attempt to apply SQI as scientific assessment tool, the research output of 33 researchers was analyzed. The study cohort was recruited from one medical university (where the authors are employed), so, in contrast to the current study, the researchers represented different disciplines of medical science but were recruited “locally” (i.e., at the same country). The methodology of analysis was quite similar to that employed in the current study. In that previous study, the author’s ranking position, determined by the *h-*index, was different to the ranking position based on SQI for the majority of the assessed scientists. Only six authors (18.2%) did not change their ranking position. Regarding the other 27 subjects, the assessment of their scientific output by SQI brought either improved (45.4%) or worse (36.4%) ranking scores. In the current study, classification figures (and positions) changed for nearly all the assessed authors. The results of both studies support the thesis that the quantity and the quality of scientific output are different ranking issues. What is important, different ranking figures, obtained from the *h-*index and SQI approach, are noticeable, regardless of the general scientific “level” of analyzed subjects. In the abovementioned study, the mean *h-*index value achieved by the researchers, recruited locally (from one country), was 15.9 ± 9.75 and for SQI 29.97 ± 21.73. In the current study, approaching the scientific outputs of globally top experts in one selected discipline, the respective values were much higher, e.g., 24.2 ± 6.2 and 92.7 ± 22.3, respectively. However, the observed relationships between the *h-*index and SQI remain similar in both groups.

Important data are shown by correlation analysis. Obviously, SQI reveals to be closer to qualitative features of research output when compared with the *h-*index.

The dynamic variability of SQI, understood as a possibility of its changes in both directions, i.e., either upwards or downwards, is its unique feature, differentiating this index from all the other traditionally used bibliometric tools. The commonly used bibliometric indices (such as the impact factor, the number of papers, the citation index, the *h-*index) are summative in character, i.e., they reflect the sum of achievements/scores of a given author in one category. A score, obtained by a given author for any of the mentioned indices, may either remain stable or increase but it will never be reduced. According to the SQI formula, when the publishing activity of a given author with a given score enters the stage, when the author’s subsequent publications are less frequently cited, then a specific “phenomenon of dilution” will occur, meaning that the more frequently cited papers will be “diluted” in a higher number of publications with low citation numbers. In consequence, the revised SQI figure will decrease. This additional and very important argument describes SQI as a bibliometric parameter, providing an effective, qualitative assessment of the current output of an individual researcher.

Further studies are necessary, involving greater groups, with different scientific outputs. Investigations in other fields of medical science, like cardiology or oncology, may reveal interesting data as well. SQI has a potential of a complementary method, possibly supporting other bibliometric tools, such as the *h-*index or the citation index.

Concluding, the qualitative features of scientific outputs, measured by SQI, changed the ranking classification of 92.5% of the assessed authors; SQI may thus be regarded as a useful tool for objective, qualitative evaluation of individual scientific output.
